# MCP-1 levels in astrocyte-derived exosomes are changed in preclinical stage of Alzheimer's disease

**DOI:** 10.3389/fneur.2023.1119298

**Published:** 2023-03-20

**Authors:** Ting Wang, Yunxia Yao, Chao Han, Taoran Li, Wenying Du, Jinhua Xue, Ying Han, Yanning Cai

**Affiliations:** ^1^Department of Biobank, Xuanwu Hospital of Capital Medical University, Beijing, China; ^2^Department of Neurobiology, Xuanwu Hospital of Capital Medical University, Beijing, China; ^3^National Clinical Research Center for Geriatric Disorders, Xuanwu Hospital of Capital Medical University, Beijing, China; ^4^Department of Neurology, The First Affiliated Hospital of Nanjing Medical University, Jangsu Province Hospital, Nanjing, China; ^5^Department of Neurology, Xuanwu Hospital of Capital Medical University, Beijing, China; ^6^Center of Alzheimer's Disease, Beijing Institute for Brain Disorders, Xuanwu Hospital of Capital Medical University, Beijing, China; ^7^Beijing Geriatric Medical Research Center, Xuanwu Hospital of Capital Medical University, Beijing, China

**Keywords:** Alzheimer's disease, astrocyte-derived exosomes, MCP-1, inflammation, immune

## Abstract

**Background:**

Alzheimer's disease (AD) is the most common form of dementia in older adults. There is accumulating evidence that inflammatory processes play a critical role in AD pathogenesis. In this study, we investigated whether inflammatory factors in plasma and astrocyte-derived exosomes (ADEs) from plasma are differentially expressed in the early stages of AD and their potential role in pathological processes in the AD continuum.

**Method:**

We included 39 normal controls (NCs), 43 participants with subjective cognitive decline (SCD), and 43 participants with amnestic mild cognitive impairment (aMCI)/AD. IL-6, IL-8, and MCP-1 in plasma and ADEs from plasma were evaluated using a commercial multiplex Luminex-based kit.

**Results:**

Pairwise comparisons between the groups showed no significant differences in plasma levels of IL-6, IL-8, or MCP-1. However, ADEs in the SCD group showed an increase in MCP-1 levels compared to the NC group. To differentiate the preclinical group, discriminant analysis was performed using sex, age, years of education, and genotype. This revealed a difference between the SCD and NC groups (area under the curve: 0.664). A Spearman correlation analysis of MCP-1 in plasma and ADEs showed no or weak correlation in the SCD (*R* = 0.150, *p* = 0.350) and aMCI/AD (*R* = 0.310, *p* = 0.041) groups, while a positive correlation in the NC group (*R* = 0.360, *p* = 0.026).

**Conclusion:**

Plasma IL-6, IL-8, and MCP-1 levels were not significantly different. However, the concentration of MCP-1 in ADEs is slightly altered during the preclinical phase of AD, which could be a potential role of the central neuron system (CNS) immune response in the AD continuum.

**Clinical trial registration:**

www.ClinicalTrials.gov, identifier: NCT03370744.

## Introduction

Alzheimer's disease (AD) is the most common form of dementia in older adults. It is a chronic, progressive, and irreversible neurodegenerative disorder (1–3). Amnestic mild cognitive impairment (aMCI) is a prodromal stage of AD. Patients with aMCI present a variety of cognitive symptoms and progress to diagnosable AD in a few years (4, 5). Subjective cognitive decline (SCD) is considered the earliest clinical presentation in the AD continuum and is defined as a perceived decline in an individual's cognitive ability relative to previous performance levels (6). Recent evidence suggests that as many as 60% of individuals with SCD progress to dementia (7).

There is increasing evidence suggesting that inflammatory processes play an important role in AD (8, 9). In the brain, neuroinflammation can cause activation of microglia, affect the normal function of astrocytes, increase pro-inflammatory factor production, and cause neuronal destruction during disease progression in AD. In the periphery, the disruption of the blood–brain barrier (BBB) permeability as well as peripheral immune cell infiltration may alter cytokine/chemokine networks in the AD brain (10). Interleukin-6 (IL-6) is a major regulator of the inflammatory response and affects neurons in both direct and indirect ways (11). Astrocytes secrete IL-6, which has multiple roles in neurodegeneration and protection and also actively regulates microglia at multiple levels during pro-inflammatory injury repair (12). Interleukin-8 (IL-8) plays a role in neutrophil trafficking and activation and is secreted by activated neutrophils (13). IL-8 is produced by astrocytes, and it has been suggested that this production may be associated with the process of cell dysfunction induced by acidosis-induced membrane destruction (14). Monocyte chemoattractant protein-1 (MCP-1) is a CC chemokine that is produced by microglia, astroglia, and neurons. In the brain, MCP-1 attracts microglia and peripheral immune cells to sites of inflammation (15). Neuroprotection can be mediated by induction and release of MCP-1 from astrocytes (16). Studies suggest that peripheral levels of IL-6, IL-8, and MCP-1 may be altered in the aMCI and AD stages (17–19). However, it is unclear whether the levels of these factors are altered in the SCD stage.

Astrocytes are neural parenchymal cells that are widely distributed in the central nervous system (CNS). Under normal physiological conditions, astrocytes maintain extracellular homeostasis, provide glucose to neurons, participate in synapse development and plasticity, and exhibit dynamic activities crucial for neural circuit function, neurological function, and behavior (20). Astrocytes have an evolutionarily ancient response to central neuron system injury and disease, commonly referred to as astrocyte reactivity (21). Under adverse conditions, reactive astrocytes can produce multiple cytokines and chemokines that can lead to neuronal degeneration, prevent BBB repair, and hinder the recovery of neurological functions (22, 23).

Recent studies show that exosomes play a key part in brain homeostasis and the crosstalk between neural cells and the periphery. In patients with AD, reactive astrocytes can cause neuroinflammatory changes through the release of inflammatory factors, cytokines, and reactive oxygen species (ROS), which can result in a redox state imbalance (24). Exosomes released by activated astrocytes appear to mediate or exacerbate AD pathological processes (25). Therefore, exosomes may contain cytokines involved in the pathogenesis of preclinical AD. However, it is unknown whether the levels of these factors are altered in astrocytes in the AD continuum.

The difference between astrocyte-derived exosomes (ADEs) and extracellular vesicles and the content of astrocyte-derived exosomes ADEs are specific to reflect the activation state of astrocytes (26), and they may provide critical information on ongoing pathological processes and may be a useful source of biomarkers of disease progression. To test these hypotheses, we used a more sensitive method (i.e., a commercial multiplex Luminex-based panel) to simultaneously detect the concentration of IL-6, IL-8, and MCP-1 in both plasma and ADEs at various clinical stages of AD.

## Materials and methods

### Participants

In the present study, a total of 125 participants were enrolled at various clinical stages, including 39 negative control (NC), 43 SCD, and 43 aMCI/AD subjects from the Sino Longitudinal Study of Cognitive Decline (SILCODE) project. SILCODE is an ongoing prospective cohort study featuring a consortium of 94 hospitals from 50 cities across China (ClinicalTrials.gov identifier: NCT03370744). This study was approved by the Xuanwu Hospital Ethics Committee, and all subjects provided written informed consent.

All 125 participants met the SILCODE project inclusion criteria. The criteria for SCD were as follows (27): (1) self-reported persistent memory decline over the last 5 years compared to the previous normal state; (2) after correcting for education level, the MMSE score is within the normal range; (3) age older than 60 years, Han nationality, and dextral; and (4) positive findings were seen on amyloid PET examination in brain regions specific to AD pathogenesis. The method was proposed by Jak and Bondi (28) to define aMCI with the following criteria: a uni- or multi-area cognitive decline with normal or mildly impaired ability to care for themselves. AD was diagnosed based on the aforementioned diagnostic criteria (29), and in addition to clinical symptom assessment, subjects underwent routine T1, T2, and FLAIR MRIs and had hippocampal atrophy. The control individuals were defined with no complaints of cognitive decline and no positive symptoms and signs on neurologic vs. general internal medicine tests, and a negative result was obtained in Aβ-PET. All participants underwent a mini-mental state examination (MMSE) and Montreal Cognitive Assessment-Basic (MoCA-B) to support the diagnosis. The exclusion criteria included brain trauma and other neurological or other systemic diseases that can cause cognitive impairment. All diagnoses were confirmed by two neurologists from Xuanwu Hospital.

### *APOE* genotyping

Leukocytes were extracted from peripheral blood using a centrifuge, and the TIANamp Genomic DNA kit (TIANGEN BIOTECH CO., LTD., Beijing, China) was used to extract genomic DNA. DNA concentration was determined with a Nanodrop 2000 spectrophotometer (Thermo Fisher Scientific, Massachusetts, USA). The *APOE* ε4 status was defined using the method of a previous report (30). The Sanger sequencing method (Sangon, Shanghai, China) was used for *APOE* genotyping.

### Extraction of ADEs from plasma

Thawed plasma was centrifuged at 12,000 × *g* for 20 min at 4°C, and a 0.25-ml aliquot of the plasma was incubated with thrombin solution, followed by the addition of calcium and magnesium-free Dulbecco's balanced salt solution (DBS^−2^) with protease inhibitor cocktail (Roche, Indianapolis, IN, USA) and phosphatase inhibitor cocktail (Thermo Fisher Scientific, Inc.). After centrifugation of thawed plasma at 3,000 × *g* for 30 min at 4°C, ExoQuick (System Biosciences, Mountain View, CA, USA) was added to the resulting supernatant and incubated for 1 h. Total exosomes were obtained and resuspended in 252.5 μl DBS^−2^ with protease inhibitor and phosphatase inhibitor cocktails. To enrich the ADEs, 0.9 μg of mouse anti-human glutamine aspartate transporter (GLAST) (ACSA-1) biotinylated antibody (Miltenyi Biotec, Inc., Auburn, CA) in 50 ml of 3% BSA (1:3.33 dilution of Blocker BSA 10% solution in DBS^−2^; Thermo Fisher Scientific, Inc.) was added into the supernatant containing total exosomes and incubated for 2 h, as described earlier (31). Thereafter, 12.5 μl of streptavidin-agarose UltraLink resin (Thermo Fisher Scientific, Inc.) in 50 μl of PBS was added and incubated for 1 h at room temperature after mixing. Centrifugation was performed at 400 × *g* at 4°C for 10 min, and then the supernatant was discarded, and 100 ml of cold 0.05 M glycine-HCl (pH 3.0) was used to resuspend the pellet by gently mixing for 10 s. the sample was incubated for 10 min and then centrifuged at 4,000 × *g* for 10 min at 4°C. Supernatants were transferred to clean tubes containing 6.5 μl of 1 M Tris-HCl (pH = 7.5) and mixed before the addition of 360 μl of mammalian protein extraction reagent (M-PER) (Thermo Fisher Scientific) with protease and phosphatase inhibitors. Then, the lysate of ADEs was stored at −80°C until an assay was performed.

### Characterization of ADEs

To calculate the concentration and average diameter of ADEs, we used nanoparticle tracking analysis (Nano Sight NS300, England). To further characterize the ADEs, transmission electron microscopy (FEI Tecnai Spirit TEM D1266, USA) was used to define the morphological structure. The ADEs were absorbed onto a 400-mesh carbon-coated copper grid (cat. no. BZ11024a; Zhongjingkeyi Technology Co., Ltd., Beijing, China), and the residual liquid was blotted with a filter paper. ADEs were stained with 2% uranyl acetate (Zhongjingkeyi Technology Co., Ltd.) for 30 s (staining repeated once) and allowed to dry for 1 min. Images were obtained on a digital camera [Gatan US4000 (895) CCD, USA].

The protein levels of common trans-membrane exosome vesicle markers were measured. Briefly, the ADEs in the M-PER-containing solution were freeze–thawed three times in liquid nitrogen and placed in a water bath to allow complete membrane lysis. Then, 5 × protein loading buffer was added to equal amounts of protein. Each sample was loaded with 20 ul and resolved on a 12% SDS-PAGE gel before transferring it onto a polyvinylidene fluoride membrane. The membrane was probed with the following primary antibodies, including positive controls: rabbit anti-CD63 antibody (EXOAB-CD63A-1, System Biosciences; 1:1,500), rabbit anti-HSP70 antibody (EXOAB-HSP70-1, System Biosciences; 1:500), mouse anti-albumin antibody (bsm-0945M, Bioss; 1:500), and glial origin-specific rabbit anti-GFAP antibody (ab7260, abcam, 1:1,0000). The negative control was rabbit anti-calnexin antibody (ET1611-86, HUABIO, 1:1,000). Antibodies were incubated at 4°C overnight.

### Measurement of inflammatory factors

Plasma was obtained by the centrifugation of blood samples at 2,500 × *g* for 10 min at 4°C within 30 min of collection using fasting morning venous blood. We tried to use commercial Human Cytokine/Chemokine/Growth Factor Panel kits (EMD Millipore Corporation, Billerica, MA, USA) to detect concentrations of IL-6, IL-8, and MCP-1 in plasma and ADEs. Low- and high-quality controls are included in the kits, and data from the plates are considered to be reliable when the sample values fall within the range indicated in the manual. Replica holes were set for each sample following the manufacturer's instructions. Luminex 200 multiplexing instrument (Luminex Corp, Austin, TX, USA) was used to read plates. The assay sensitivities for IL-6, IL-8, and MCP-1 were 0.2, 0.58, and 3.24 pg/ml, respectively. Because differences in ADE recovery rates among subjects remained, and CD81 as a member of tetraspanins, always used as an internal control in most studies. In this study, CD81 in ADEs was also measured by using human CD81 antigen ELISA kits (Wuhan Huamei Biotech Co., Ltd., Wuhan, China). The mean value of CD81 in each assay group was set at 1.00, and relative values of CD81 for each sample were used to normalize their recovery (32).

### Statistical analysis

Statistical analyses were performed using SPSS V.22.0 (IBM Corp, New York, NY, USA) and R. The Shapiro–Wilk test was used to assess data distribution normality. The *x*^2^ test was used to analyze distributions of sex and *APOE* genotype. Tukey's test was used to analyze age. Dunn's test was used to analyze the MMSE score, MoCA-B score, and years of education. Differences in inflammation factors of plasma and ADEs levels between the various groups were further verified using univariate linear regression after correcting for confounding factors, including age, sex, APOE genotype, years of education, and MCP-1 levels in ADEs/plasma. The diagnostic ability of indicators to distinguish the SCD group from the NC group was evaluated using receiver operating characteristics (ROC) curve analysis. To examine whether correlations existed between the levels of MCP-1 in plasma and ADEs, Spearman correlation coefficients were calculated. All tests were two-tailed, and the significant difference was set to a *p*-value of < 0.05.

## Results

### Demographic data

Demographic and clinical information are shown in Table 1. A total of 125 participants were included in this study, including 39 normal controls (NCs), 43 patients with SCD, and 43 patients with aMCI/AD. Although there was no significant difference in age or sex between the three groups, we could observe that the aMCI/AD group had a higher age than the other two groups, and a higher proportion of women had aMCI/AD, which is consistent with earlier findings (33, 34). MMSE scores and MoCA-B scores did not pass the Shapiro–Wilk test (*p* < 0.05) and show significant differences between various groups. The frequency of *ApoE* ε4 allele carriers (Table 1) in different groups also observed significant differences.

**Table 1:** Demographic and clinical characteristics of subjects.

	**NC**	**SCD**	**aMCI/AD**	***p*-value**
*N* (125)	39	43	43	
Sex (F/M)	22/17	32/11	25/18	0.168
Age, years	66.97 ± 4.95	67.12 ± 5.81	70.03 ± 8.88	0.112
Years of education	12.00 [11.00–16.00]	12.00 [11.00–15.00]	12.00 [9.00–15.00]	0.329
MMSE scores	29.00 [28.00–30.00]	29.00 [28.00–30.00]	23.00 [19.50–25.00]	0.000
MoCA-B scores	26.00 [24.00–28.00]	27.00 [24.00–28.00]	17.00 [14.00–21.00]	0.000
*APOE* ε4 carriers (%)	38.46	27.91	60.47	0.008

Age is presented as mean ± standard deviation (SD). Years of education, MMSE, and MoCA-B scores are presented as median (interquartile range [IQR]). NC, normal controls; SCD, subjective cognitive decline; aMCI, amnestic mild cognitive impairment; AD, Alzheimer's disease; MMSE scores, mini-mental state examination scores; MoCA-B, scores Montreal Cognitive Assessment-Basic scores.

### Characterization of ADEs in plasma

**Figure 1:**
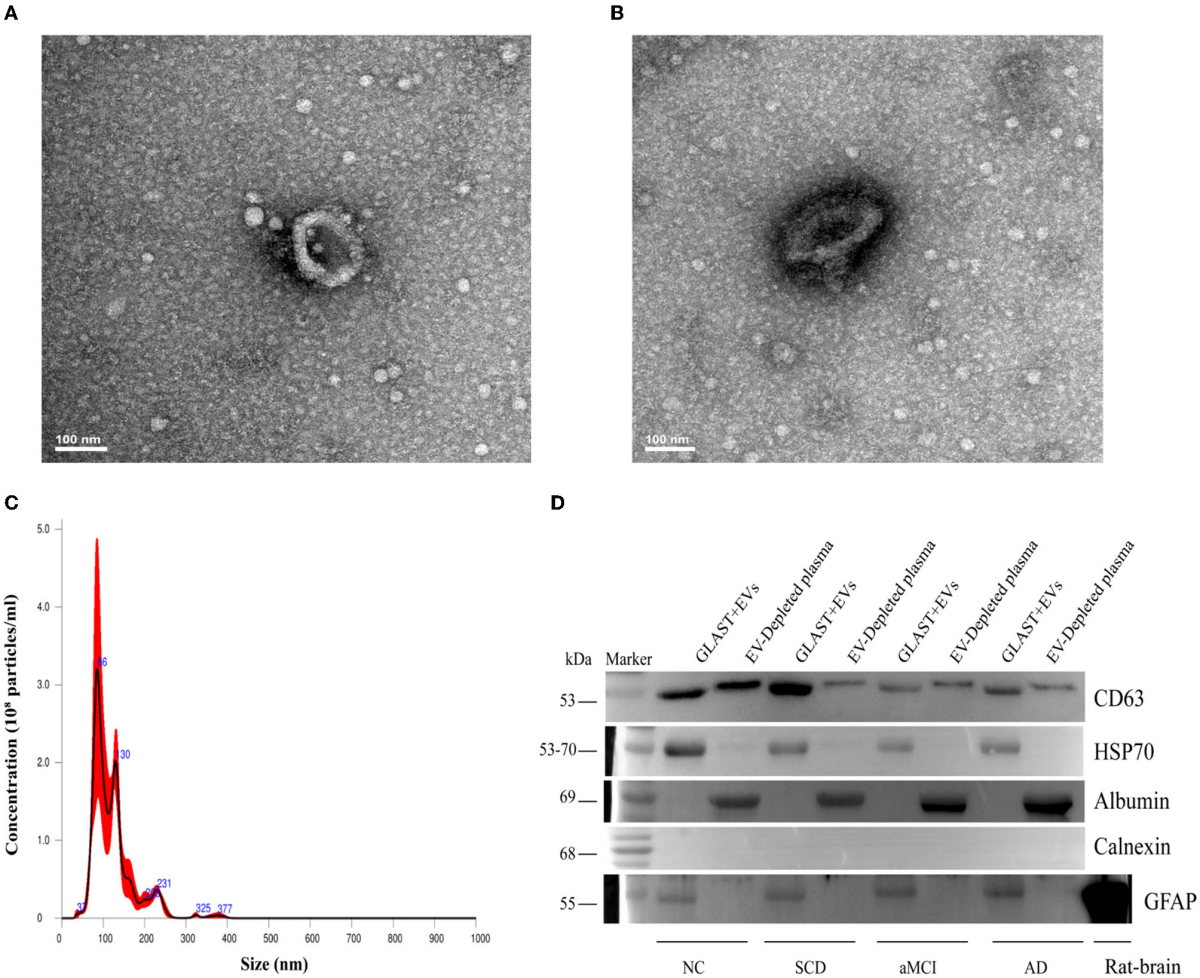
Characterization of ADEs. **(A, B)** Typical TEM images of ADE. Characteristic image of ADE from one subject and the analysis was performed at Tsinghua University. **(C)** Typical NTA results. The graphs display particle concentration (particles/ml) against the size (diameter in nm) of one subject. **(D)** Western blot characterization of ADEs. Western blots of astrocyte-enriched (GLAST+) plasma EVs compared to EV-depleted plasma (supernatant obtained after Exoquick^®^) from four subjects. First, compared with the supernatant, ADEs had obviously higher CD63 and HSP70 levels, demonstrating the nature of EVs. Second, compared to the supernatant, ADEs showed no albumin levels. In addition, Calnexin was not clearly observed in ADEs, suggesting purity. Third, GFAP, a classic astrocyte marker was clearly observed in ADEs, suggesting a true astrocyte origin, and rat-brain as a positive control. TEM, transmission electron microscope; ADEs, astrocyte-derived exosomes; NTA, nanoparticle tracking analysis; GLAST, glutamine aspartate transporter; NC, normal controls; SCD, subjective cognitive decline; aMCI, amnestic mild cognitive impairment; AD, Alzheimer's disease.

ADEs from plasma were evaluated by TEM and Nanosight to characterize morphology and size distribution. ADEs displayed a typical cup-shaped structure with a diameter of 100 nm (Figures 1A, B). Nanosight showed that the particles had an average diameter of 123.9 ± 5.6 nm, and the concentration was 1.87e + 08 +/– 5.01e + 07 particles/ml, within the range of diameters of exosomes (Figure 1C). Western blot analysis of one NC and three patients demonstrated that CD63, a membrane protein, HSP70, a cytosolic marker, and GFAP, an astrocyte-specific marker, were enriched, while calnexin, a negative marker, was absent in ADEs. Furthermore, albumin, a major component of non-EV co-isolated markers, was enriched in EV-depleted plasma (Figure 1D, the full pattern of each protein by Western blot is shown in Supplementary Figures 1–5). These results indicate that the ADEs purified in the present study are similar in morphology, size distribution, and surface markers to those previously reported (35).

### Inflammatory factor expression in the different clinical groups

**Figure 2:**
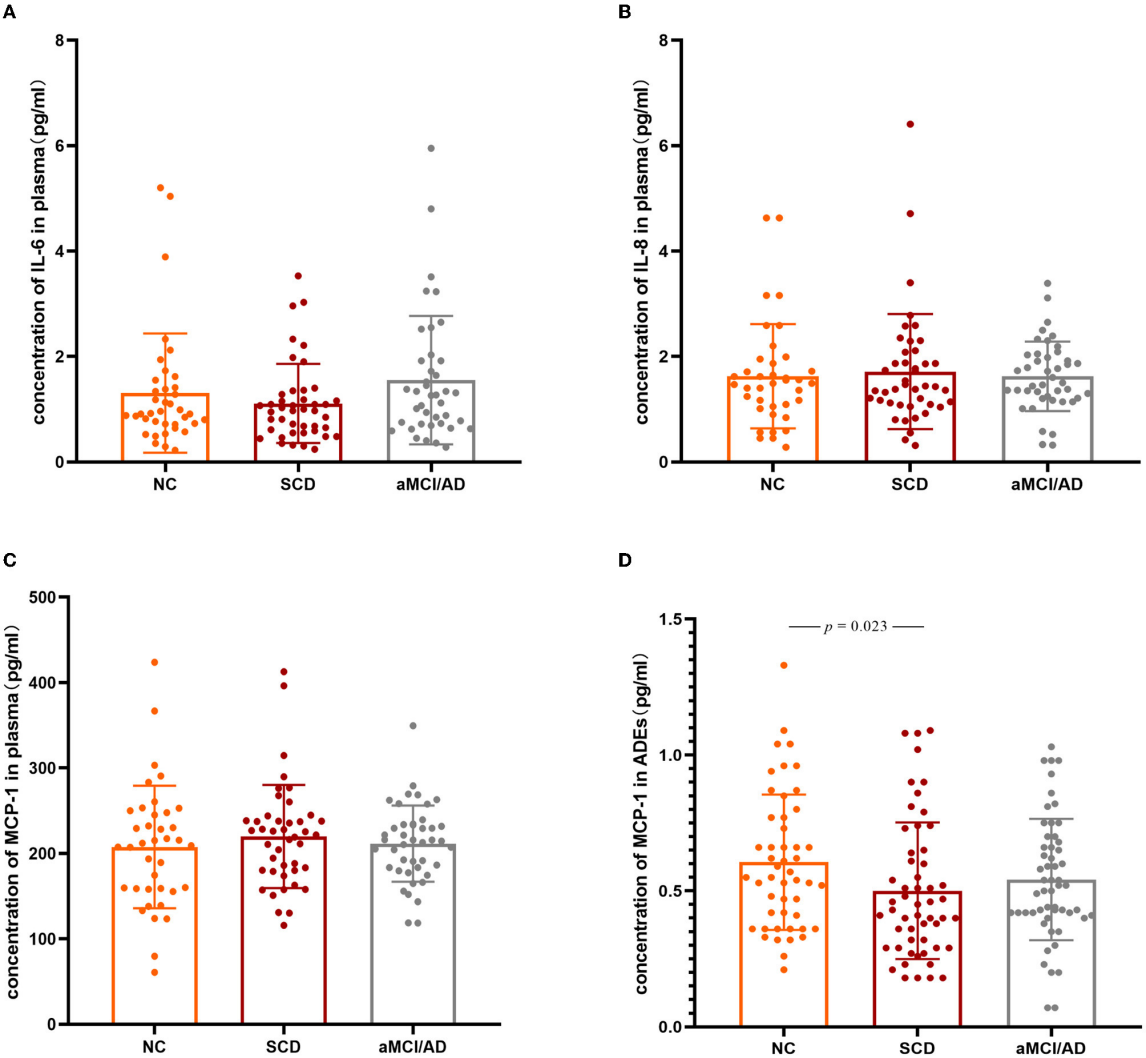
Three inflammation factors in the NC, SCD, and aMCI/AD groups. The scatter plots with SD present the concentrations of plasma IL-6 **(A)**, IL-8 **(B)**, MCP-1 **(C)**, and MCP-1 levels in ADEs (after normalized with CD81) **(D)** in different clinical groups. The statistically significant *p*-values were marked. IL-6, Interleukin-6; IL-8, Interleukin-8; MCP-1, monocyte chemoattractant protein-1; NC, normal controls; SCD, subjective cognitive decline; aMCI, amnestic mild cognitive impairment; AD, Alzheimer's disease.

IL-6, IL-8, and MCP-1 were all detected in plasma. No significant differences were observed in plasma IL-6, IL-8, or MCP-1 between the groups (Figures 2A–C), while sex, age, years of education, *APOE* ε4 status, and MCP-1 in plasma as covariants. The analysis results without the covariants are shown in Supplementary Figure 6. MCP-1 was detected in ADEs, while IL-6 and IL-8 were not. MCP-1 levels in ADEs were significantly decreased in patients with SCD compared with the NC group (*p* = 0.023) (Figure 2D). None of those inflammatory factors passed the normality test (*p* < 0.05). We also analyzed the effect of carrying or not carrying the *ApoE4* gene on the expression of inflammatory factors, but no significant differences were found (*p* > 0.05).

### Developing a model to distinguish patients with SCD from NCs

**Figure 3:**
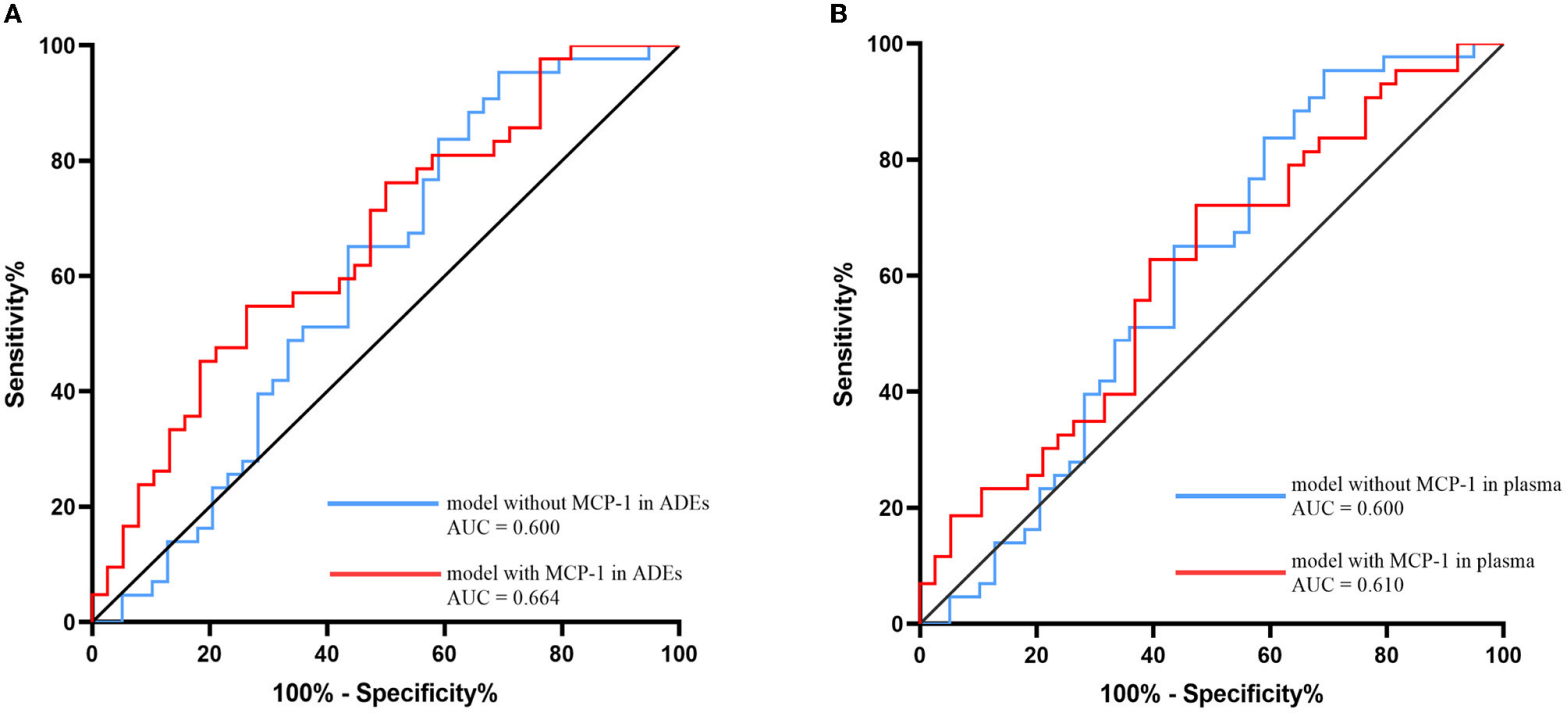
Receiver operating characteristic (ROC) evaluated the MCP-1 in ADEs **(A)** or MCP-1 in plasma **(B)** ability to distinguish the SCD and NC group. Models with sex, age, APOE genotype, and years of education included as covariates with MCP-1 in ADEs/plasma are shown with red lines and without MCP-1 in ADEs/plasma are shown with blue lines. MCP-1, monocyte chemoattractant protein-1; ADEs, astrocyte-derived exosomes; NC, normal controls; SCD, subjective cognitive decline.

ROC analysis was performed to assess the ability of the model to distinguish participants with SCD from NCs, including sex, age, *APOE* genotype, and years of education. In the model with ADEs-included MCP-1, the discriminatory power was relatively strong (area under the curve (AUC): 0.664; 95% CI 0.545–0.782; *p* = 0.001). In addition, the model with MCP-1 in the plasma was tested (area under the curve (AUC): 0.610; 95% CI 0.486–0.733; *p* = 0.090). The model did not include MCP-1 in ADEs or plasma, and the discriminatory power was relatively weak (AUC: 0.600; 95% CI 0.473–0.727; *p* = 0.120; Figure 3). Thus, the model with MCP-1 in ADEs had a moderate ability to predict the SCD stage.

### Correlation analysis of the inflammation factors

**Figure 4:**
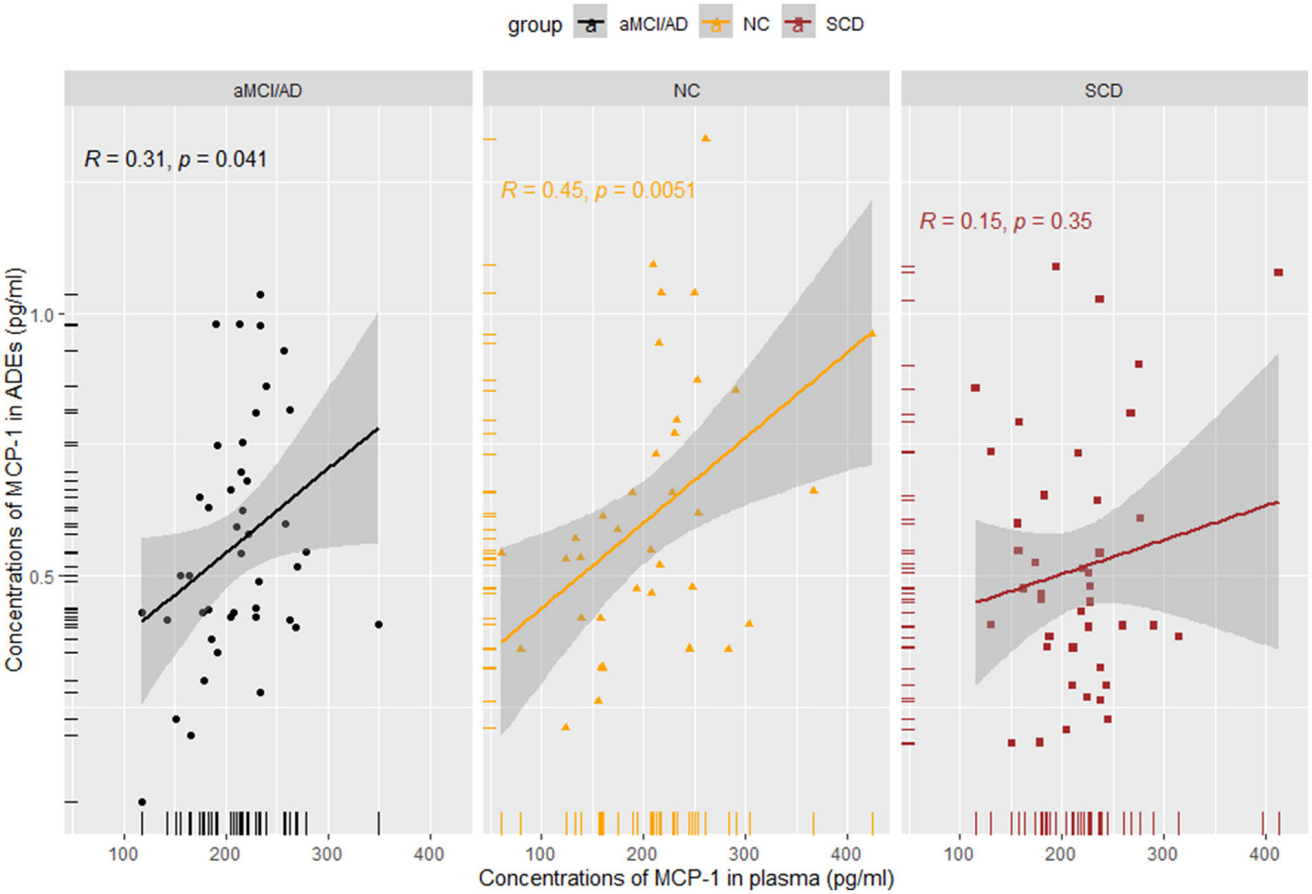
Spearman rank correlation scatterplots between MCP-1 in plasma and MCP-1 in ADEs in different groups. A weaker correlation was obtained in the SCD and aMCI/AD groups (SCD: *R* = 0.15, *p* = 0.35; aMCI/AD: *R* = 0.31, *p* = 0.041).

MCP-1 levels in ADEs were positively correlated with those in the plasma in NCs (*R* = 0.45, *p* = 0.0051) and in patients with aMCI/AD (*R* = 0.310, *p* = 0.041). No correlation was identified in SCD subjects (*R* = 0.150, *p* = 0.350) (Figure 4). We also performed a correlation analysis between IL-6 and IL-8 in plasma and MCP-1 in ADEs, but no significant association was observed (Supplementary Figure 7). However, strong positive correlations were observed between plasma IL-6, IL-8, and MCP-1 in the NC group (*p* < 0.01), and also found a strong positive correlation between MCP-1 and IL-8 in the plasma of patients with SCD (*R* = 0.53, *p* = 0.00025); the detailed results are presented in the Supplementary Figure 8. Unfortunately, we did not observe meaningful results in the analysis of the correlation between MCP-1 in ADEs and the degree of cognitive impairment (Supplementary Figure 9).

## Discussion

Accumulating evidence suggests that chronic inflammation may greatly influence the pathogenesis of AD (36). Cytokine levels in the peripheral blood of patients with AD have been investigated in numerous studies; however, reliability and clinical usefulness have been limited. In neurodegenerative diseases, ADEs have been suggested to play an important role in propagating neuropathology or exacerbating the degree of neurodegeneration (37), and because of the wide range of neural cargo proteins in ADEs isolated from plasma samples, a growing number of studies have focused on the role of exosomes in AD pathogenesis (38–41). In this study, the expression of inflammatory factors in plasma and ADEs were measured at different phases of AD. Notably, the level of MCP-1 was significantly different in ADEs at the preclinical stage of AD. To our knowledge, this study is the first to measure MCP-1 in plasma and ADEs simultaneously in patients with SCD.

The levels of IL-6, IL-8, and MCP-1 have been extensively studied in the peripheral blood of patients with AD. A meta-analysis of 40 studies comprising 2,295 patients with AD revealed significantly elevated IL-6 concentrations compared with healthy individuals but with significant heterogeneity in peripheral blood (42). Rufina et al. found no significant changes in IL-6 or IL-8 in the serum of patients at different stages of AD recruited from multiple regions (43). In another meta-analysis of 540 patients with AD, the levels of MCP-1 in peripheral blood showed no difference compared with healthy individuals (44). Janelidze et al. (45) also did not observe any significant change in IL-6, IL-8, or MCP-1 levels between AD and controls using the MSD method (45). These studies suggest that plasma IL-6, IL-8, and MCP-1 may not differ between AD and normal subjects. While our study is consistent with these studies, Lee et al. (46) study with more patients and 2 years of follow-up found higher plasma MCP-1 levels in patients with AD compared to patients with MCI and controls (46), and their finding was consistent with another study that included a large cohort of patients with AD (19). The reason why no changes in plasma MCP-1 levels were found in our study may be due to the relatively small number of patients with AD. Therefore, the variability of MCP-1 in the plasma during the disease phase of AD requires more studies with large sample sizes to confirm. Our study suggests that there is no difference in the expression of those cytokines in the plasma during the SCD phase of AD.

It is well known that the periphery and the brain can communicate *via* the BBB and that neuroinflammation is a hallmark of AD. Recent studies show that glial-related pathways are central to AD risk and pathogenesis (47, 48). Reactive astrocytes surround amyloid plaques and secrete various pro-inflammatory cytokines and are considered to have an early, primary role in AD progression. In our study, MCP-1 concentrations in ADEs were significantly lower in patients with SCD compared to NC subjects. The aberrant oligomeric Aβ in AD may lead to extensive reactive astrogliosis and the dysregulation of numerous homeostatic processes (49). Astrocytes can release EVs that contribute to the development of neuroinflammatory and neuropathological disorders (50). It was reported that MCP-1 released from astrocytes can reduce neuronal damage (16, 51), and in animal models of AD, it plays a major role in plaque clearance and macrophage recruitment (52), suggesting a neuroprotective role in the CNS. Notably, a consequence of MCP-1 downregulation may be the induction of neurodegeneration as an immune imbalance may result in neurodegeneration and cognitive decline. Thus, the reduced levels of MCP-1 in patients with SCD, while a tendency to increase during the clinical phase of AD, maybe because the SCD stage was the initial onset and the immune system could not respond in a timely manner, whereas in the disease stage, the immune system adjusted the response state. Unfortunately, we did not detect IL-6 or IL-8 in ADEs. In contrast to MCP-1, IL-6, and IL-8, secreted proteins are expressed at low levels and using a Meso Scale Discovery (MSD) method might detect the low abundance of proteins in ADEs from plasma (53). Therefore, a more sensitive method might facilitate the detection of proteins in plasma exosomes.

We performed an analysis of the correlation between MCP-1 in ADEs and MCP-1 in plasma. A stronger correlation was observed between MCP-1 in plasma and ADEs under normal physiological conditions, whereas a weaker correlation was observed between MCP-1 in plasma and ADEs at the time of cognitive decline and clinical symptoms, possibly suggesting that immune responses in the brain take precedence over those in the periphery. Thus, ADEs may rapidly and sensitively reflect central pathological changes. No significant associations were observed with inflammatory factors in the plasma during the disease stage of AD presumably due to their different roles in the pathological mechanisms or the relatively small sample size of the group.

A direct reflection of the state of the CNS *via* CNS-derived exosomes is considered (53). Therefore, the expression of MCP-1 in CNS-derived exosomes, as compared to blood or CSF, may better shed light on the role of MCP-1 in AD. Recently, Jia et al. (54) revealed that measuring exosomal Aβ_42_, T-tau, and P-T181-tau from blood might be a useful way to diagnose aMCI and AD (54). Furthermore, Charisse et al. reported that complement protein levels in ADEs may be predictive biomarkers of MCI conversion to AD (32). Thus, proteins in exosomes may have great potential for the prediction of AD and related diseases.

There are some limitations to our study. First, the sample size is relatively small, and the aMCI and AD subgroups were pooled together as one group, which may have negatively impacted statistical power, such as the power of MCP-1 in ADEs to discriminate SCD from NC or the relevance of MCP-1 in ADEs to cognitive function. Second, all subjects originated from the same region, which could have potentially led to geographic bias. Third, neuropsychological tests were relied primarily on for the diagnosis of SCD and aMCI. As a result, misdiagnosis may not be adequately avoided. Fourth, this study was a cross-sectional study. Future longitudinal multi-center studies may help us better understand the dynamic changes in inflammatory factors during AD progression. Finally, the *APOE* ε4 carriers in NC were slightly higher compared to other studies. Although we inquired about the lifestyle of the participants, the inclusion and exclusion criteria did not specify the lifestyle; a study showed a wide variation in *APOE* ε4 carrier rates in different lifestyles (55); thus, detailed lifestyle segmentation at enrollment may reduce bias in *APOE* ε4 carrier rates in normal populations.

Here, concentrations of IL-6, IL-8, and MCP-1 were examined in both plasma and ADEs. Our findings suggest that MCP-1 levels in ADEs changed during the early stage of AD, and therefore, it may be a potential role of CNS immune response in the AD continuum.

## Conclusion

Plasma levels of IL-6, IL-8, and MCP-1 were not significantly different, but the concentration of MCP-1 in ADEs was slightly altered in the preclinical phase of AD.

## Data availability statement

The raw data supporting the conclusions of this article will be made available by the authors, without undue reservation.

## Ethics statement

This study was approved by the Xuanwu Hospital Ethics Committee, and all subjects provided written informed consent.

## Author contributions

YC designed the study. TL, WD, and YH recruited participants and performed the clinical investigation. TW and YY performed the extraction of ADEs from plasma and the characterization of ADEs. TW and JX genotyped *APOE*. TW performed Luminex assay. TW and CH performed the data analysis and wrote the manuscript. All co-authors contributed to revising the manuscript for intellectual content and approved the final version for publication.
