# Supplementary material for: MCP-1 levels in astrocyte-derived exosomes are changed in preclinical stage of Alzheimer's disease
Source: Front Neurol. 2023 Mar 20;14:1119298. doi: 10.3389/fneur.2023.1119298 (PMC10067608; doi:10.3389/fneur.2023.1119298)
Supplement: Supplementary file 1 [file Data_Sheet_1.docx]

Supplementary Information for

**MCP-1 levels in** **astrocyte-derived exosomes are changed in preclinical stages of Alzheimer’s disease**

Ting Wang^a,b,#^, Yunxia Yao^a,b,#^, Chao Han^c,#^, Taoran Li^d^, Jinhua Xue^a,b^, Ying Han^b,c,g,*^, Yanning Cai^a,b,c,f,*^

*Correspondence: Yanning Cai, Address: Department of Neurobiology, Xuanwu Hospital, Capital Medical University, 45 Changchun Street, Beijing 100053, China. E-mail: [yanningcaimailbox@163.com](mailto:yanningcaimailbox@163.com); Tel.: +861352259695; Fax: +86 (010)83161294

*Correspondence: Ying Han, Address: Department of Neurobiology, Xuanwu Hospital, Capital Medical University, 45 Changchun Street, Beijing 100053, China. E-mail: [hanying@xwh.ccmu.edu.cn](mailto:hanying@xwh.ccmu.edu.cn); Tel.: +86 13621011941; Fax: +86 (010)83167306


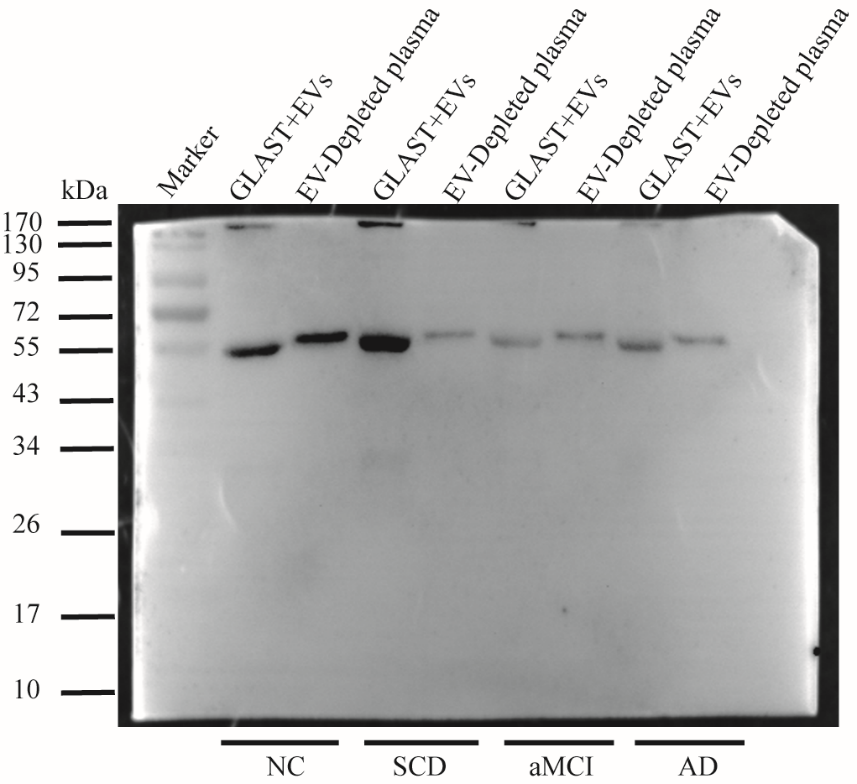


Supplementary Figure 1. Western blot of CD63 in astrocyte derived exosomes and EV-Depleted plasma in NC, SCD, aMCI, and AD subjects.


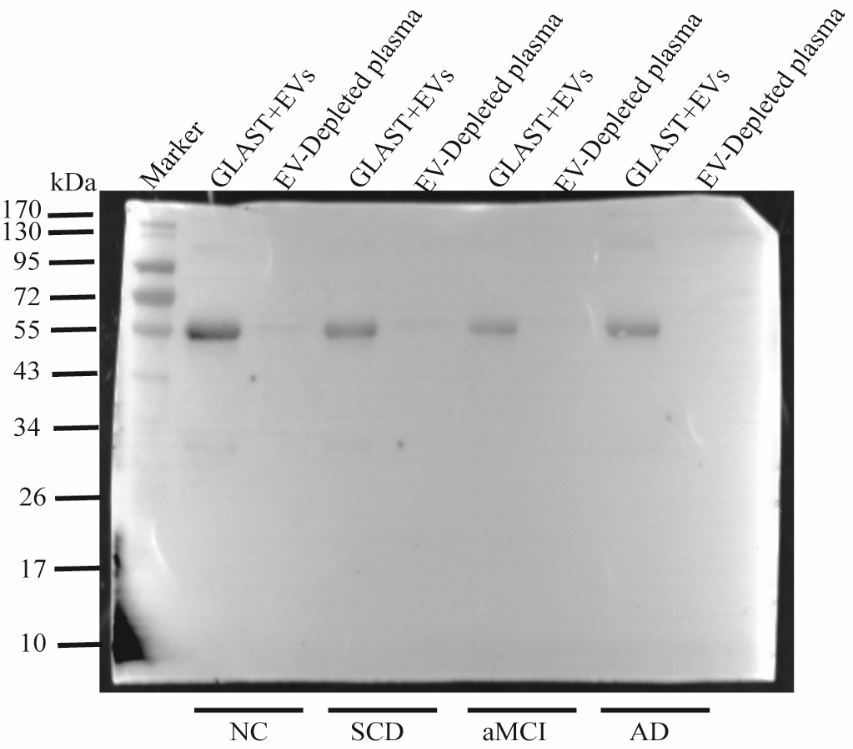


Supplementary Figure 2. Western blot of HSP70 in astrocyte derived exosomes and EV-Depleted plasma in NC, SCD, aMCI, and AD subjects.


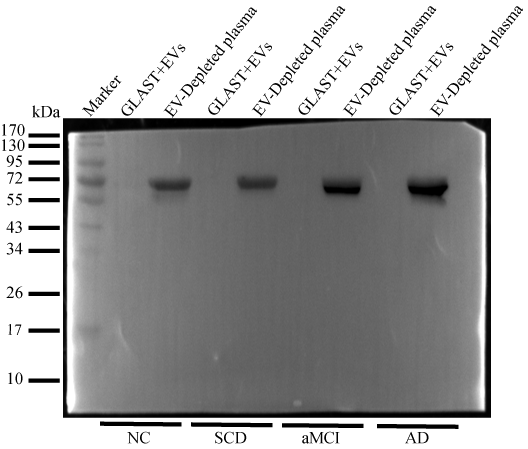


Supplementary Figure 3. Western blot of Albumin in astrocyte derived exosomes and EV-Depleted plasma in NC, SCD, aMCI, and AD subjects.


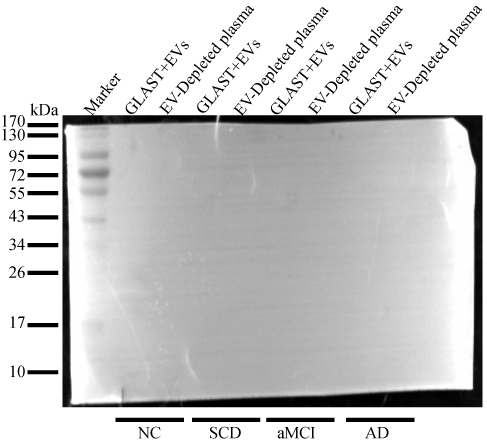


Supplementary Figure 4. Western blot of Calnexin in astrocyte derived exosomes and EV-Depleted plasma in NC, SCD, aMCI, and AD subjects.


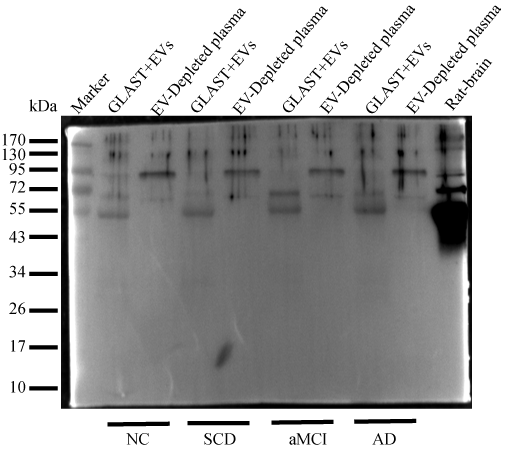


Supplementary Figure 5. Western blot of GFAP in astrocyte derived exosomes and EV-Depleted plasma in NC, SCD, aMCI, and AD subjects, Rat-brain as the positive control.


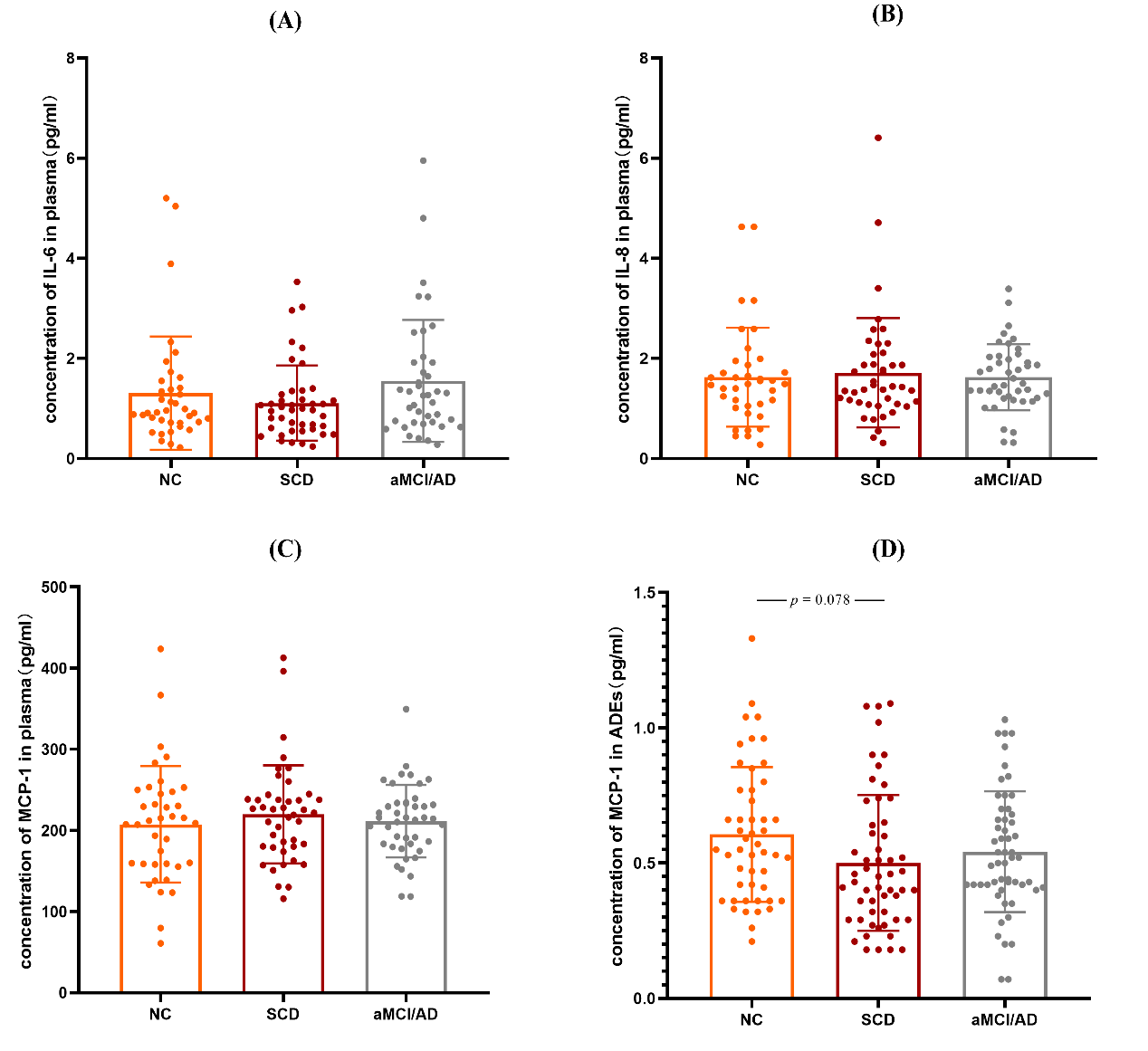


Supplementary Figure 6. Three inflammation factors in the NC, SCD, and aMCI/AD groups. The scatter plots with SD were present the concentrations of plasma IL-6 (A), IL-8 (B), MCP-1 (C) and MCP-1 levels in ADEs (after normalized with CD81) (D) in different clinical groups. ​The *p*-values of MCP-1 in ADEs was marked.


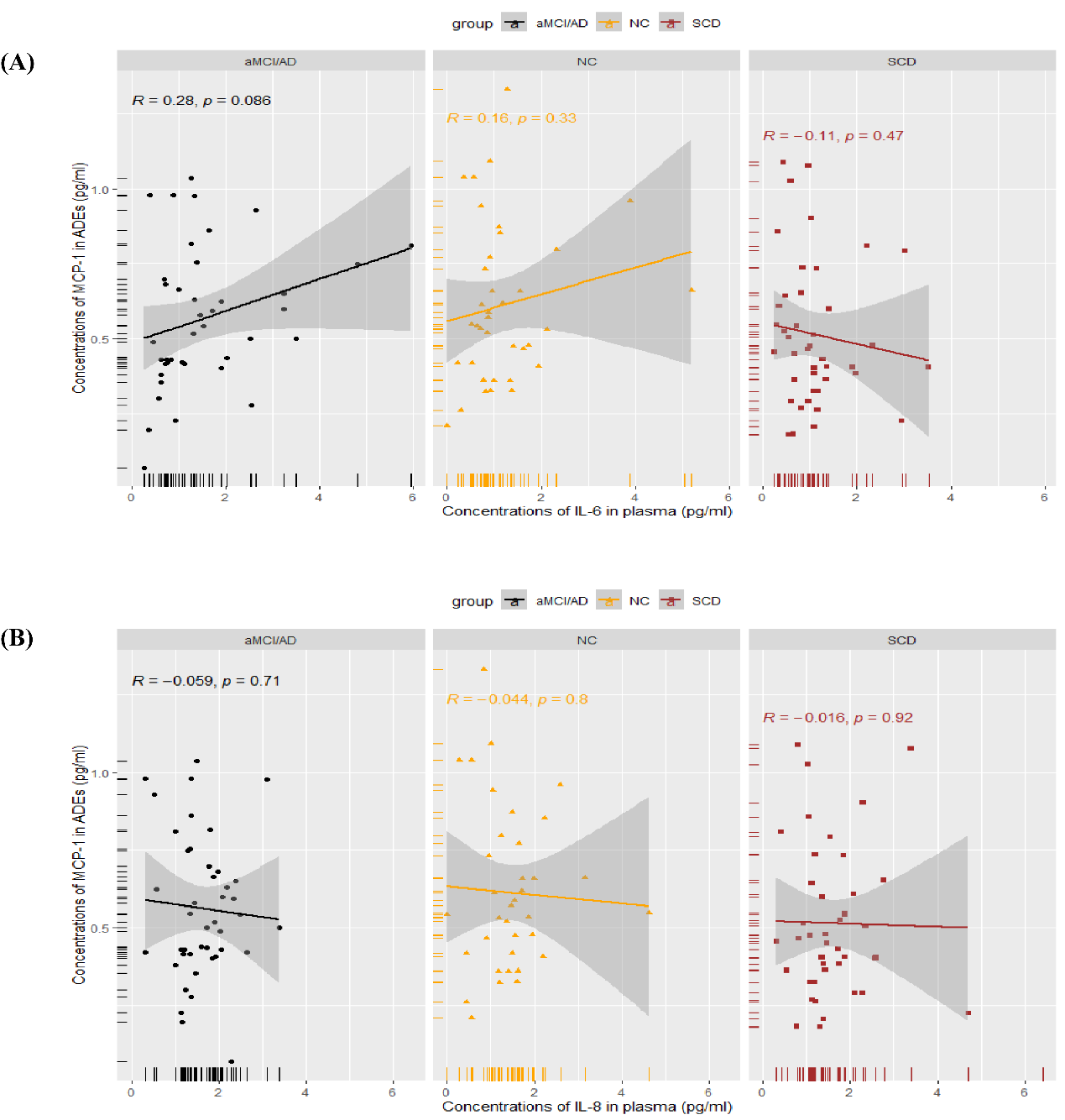


Supplementary Figure 7. Spearman rank correlation scatterplots between IL-6 (A) and IL-8 (B) in plasma and MCP-1 in ADEs.


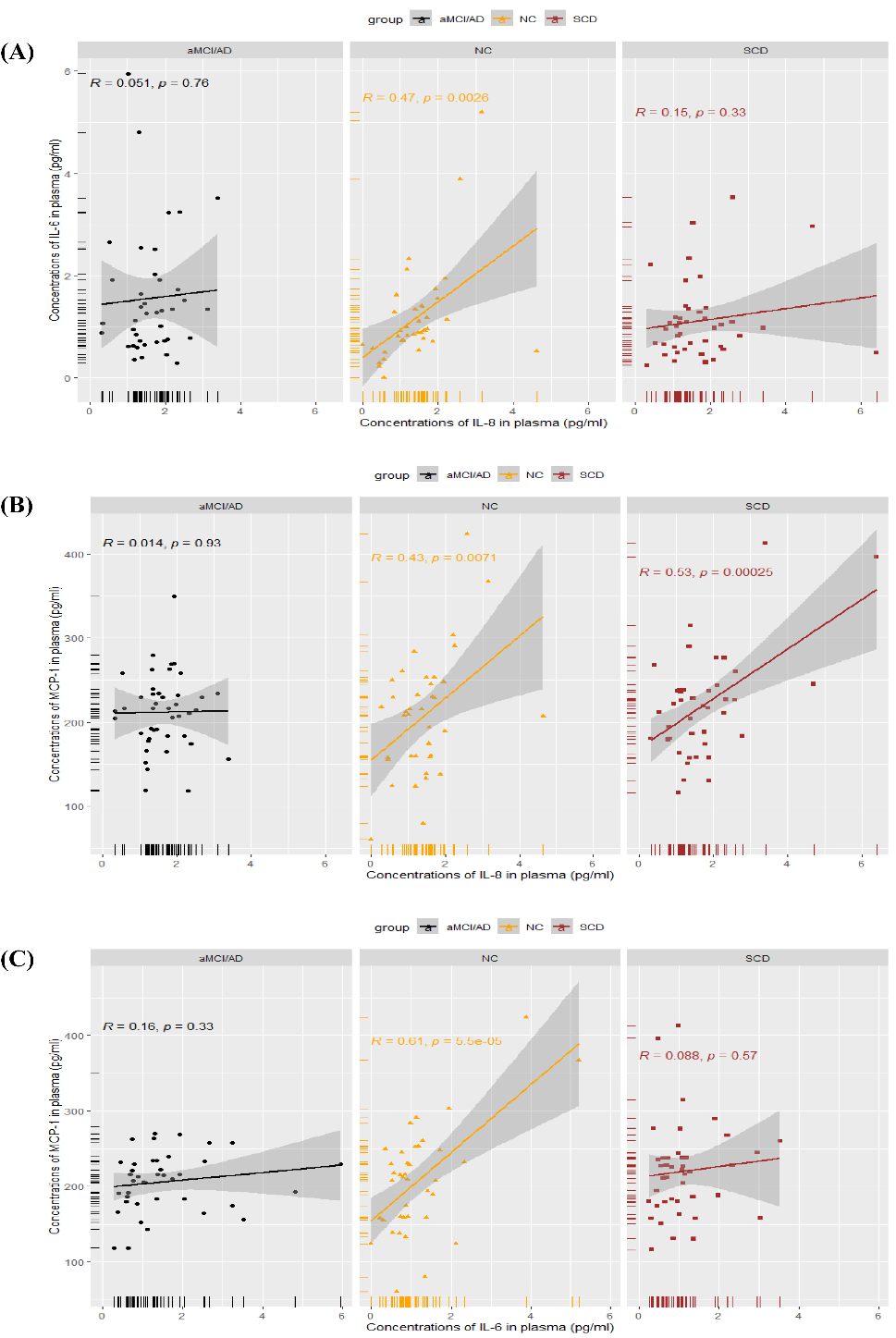


Supplementary Figure 8. Spearman rank correlation scatterplots between IL-6, IL-8 and MCP-1 in plasma.


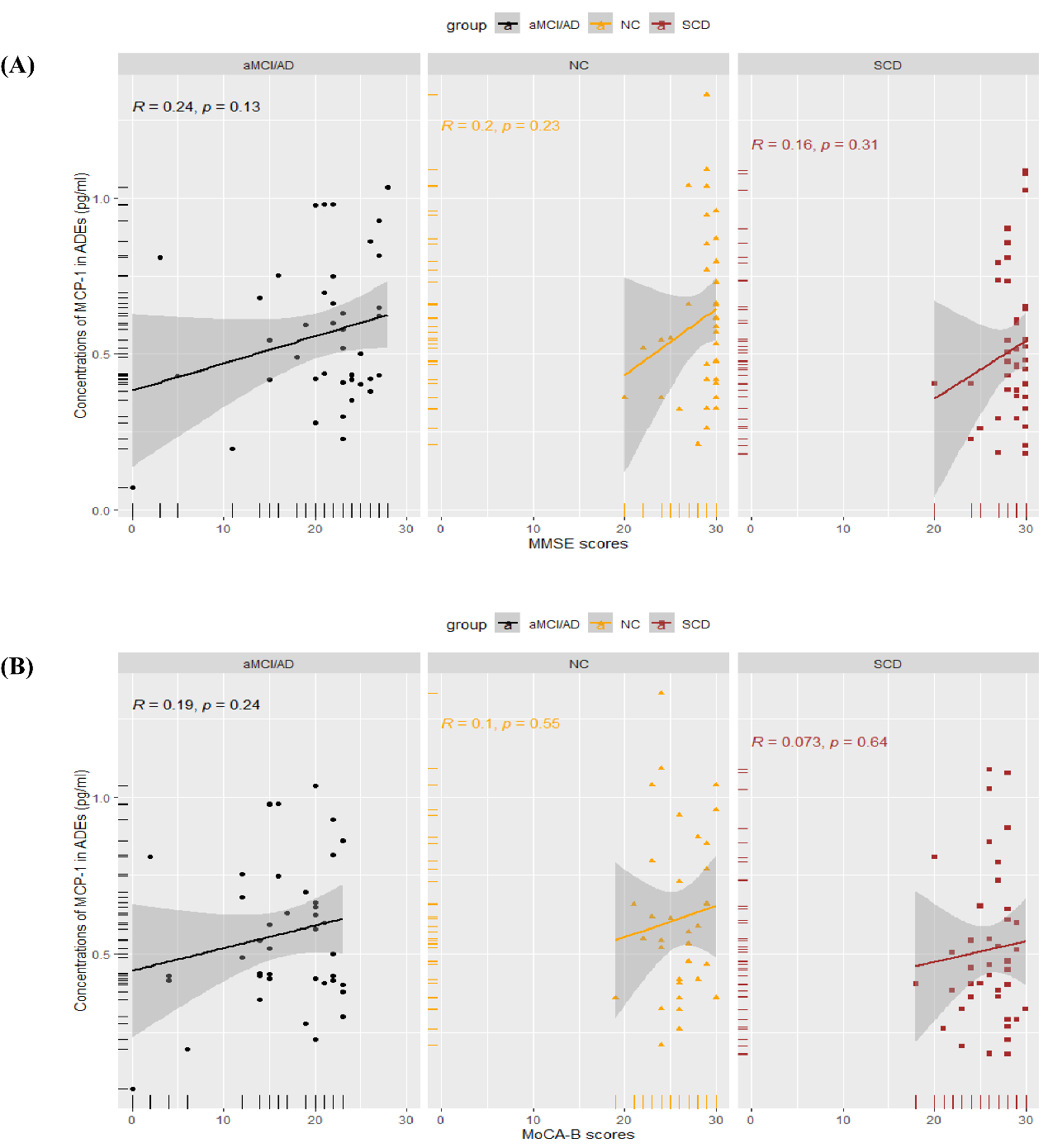


Supplementary Figure 9. Spearman rank correlation scatterplots between MMSE scores (A), MoCA-B scores (B) and MCP-1 in ADEs.
